# Multi-objective pathfinder algorithm for multi-objective optimal power flow problem with random renewable energy sources: wind, photovoltaic and tidal

**DOI:** 10.1038/s41598-023-37635-7

**Published:** 2023-06-30

**Authors:** Ning Li, Guo Zhou, Yongquan Zhou, Wu Deng, Qifang Luo

**Affiliations:** 1 College of Artificial Intelligence, Guangxi University for Nationalities, Nanning, China; 2 Department of Science and Technology Teaching, China University of Political Science and Law, Beijing, China; 3 Xiangsihu College of Guangxi University for Nationalities, Nanning, China; 4 College of Electronic Information and Automation, Civil Aviation University of China, Tianjin, China; 5 Guangxi Key Laboratories of Hybrid Computation and IC Design Analysis, Nanning, China; 6 Computer Science School, Wuhan University, Wuhan, China

**Keywords:** Energy science and technology, Engineering

## Abstract

In this paper, the multi-objective optimal power flow (MOOPF) problem optimization objectives focus on four optimization objectives: generation cost, emission, real power loss and voltage deviation (VD). Three renewable energy sources with successful industrial applications, including wind energy, solar energy, and tidal energy are introduced. Renewable energy supply is uncertain, so Weibull distribution probability, lognormal probability and Gumbel probability are used to calculate the instability and intermittency of wind energy, solar energy and tidal energy, respectively. The inclusion of four energy supplies on the IEEE-30 test system and the consideration of renewable energy reserves and penalty cost calculation improve the realism of the model. In order to obtain the control parameters that minimize the four optimization objectives, a named multi-objective pathfinder algorithm (MOPFA) based on elite dominance and crowding distance was proposed to solve this multi-objective optimization problem. Simulation results show the feasibility of the model, and MOPFA can get more evenly distributed Pareto front and provide more diverse solutions. A compromise solution was selected by the fuzzy decision system. Comparison with the recently published literature also shows that the proposed model can effectively reduce emissions and other indicators. In addition, the statistical test results show that MOPFA's multi-objective optimization performance ranks first. In solving this complex optimization problem, results show the MOPFA is superior to other multi-objective algorithms in optimization accuracy and speed.

## Introduction

The stability of the electric power system, which serves people's production and lives, is an important problem. It is well worth studying how to optimize the parameters in electric control system. Optimal Power Flow (OPF) is an important tool for optimizing the power system, which is important for the reliable operation and cost reduction of the power system^1^. The OPF problem is characterized by nonlinearity and multiple constraints, these constraints include generator capability, line capacity, bus voltage and power flow balance^2^. The OPF problem belongs to the NP-hard problem, which adds to the difficulty of searching for the solution^3^. The goal of optimization is to find the optimal solution that minimizes the objective in the solution set of many control variables to be selected. Researchers usually consider the optimization of a single objective, such as the total fuel cost of generators, power loss, emissions and other objectives. However, reducing power loss should also reduce generator costs or other cost targets, therefore, multiple optimization objectives should be considered in the OPF problem, but unfortunately these goals are in conflict. The OPF problem of using thermal generators for power output has long been considered. On the other hands, with the widespread application of renewable energy, the share of renewable energy (e.g., wind power, solar power and tidal power) in the power system is increasing. It becomes necessary to study the multi-objective OPF problem with the grid connection and uncertainty characteristics of renewable energy sources^4^.

In this study, we focus on the MOOPF problem with integrating renewable energy supply. This research considers the grid connection with wind, solar and tidal energy, Weibull distribution, Lognormal and Gumbel probability density functions are used to calculate the uncertainty of wind, solar and tidal energy, respectively^5^. The objective of optimization is to obtain the minimum values of fuel costs, emissions, real power loss and voltage deviation at the same time. However, the MOOPF problem itself has to deal with multiple conflicting objective functions, coupled with many nonlinear constraints, which greatly increases the complexity and difficulty of solving the MOOPF problem. To better solve this problem, a named multi-objective pathfinder optimization algorithm (MOPFA) is proposed, MOPFA is a multi-objective metaheuristic algorithm based on non-dominated sorting, crowded distance and elite archiving components. Elites correspond to pathfinder individuals in PFA groups, which can better lead followers to search. MOPFA is applied to solve a multi-objective OPF problem with wind and solar energy. The IEEE 30-bus system^6^ was modified to integrate multiple renewable energies. Experiments are carried out on a modified IEEE 30-bus system. The experimental results obtained by MOPFA were compared with those of powerful multi-objective optimizers, and MOPFA ranked first in performance metrics. Compared with the models proposed in recent published literature, this paper proposed a MOOPF problem model for hybrid wind, solar and tidal energy that can effectively reduce emissions and achieve other optimization objectives.

The main contributions of this study can be summarized as follows:

- A novel multi-objective pathfinder optimization algorithm (MOPFA) is proposed based on non-dominated sorting, crowded distance and elite archiving components.
- The multi-objective OPF problem with wind, solar and tidal energy was studied, while the uncertainties of renewable energy were studied. The widely known IEEE 30-bus was modified to include renewable energy systems, and MOPFA used it as a test system to solve the MOOPF problem.
- Experiments show MOPFA obtains a more uniform Pareto front to provide more diverse solutions, while MOPFA's compromise solution can reduce pollution emissions while reducing costs and other indicators.

The rest of this study is organized as follows. Section "Related work" summarizes related studies. Section "Mathematical models" introduces the MOOPF problem with wind, solar and tidal energy to formulate a MOOPF problem model with renewable source. Section "Uncertainty and power models for renewable energy" explains the uncertainty of a renewable source. In section "Multi objective pathfinder optimization algorithm (MOPFA)", the proposed multi-objective pathfinder algorithm (MOPFA) is introduced and applied to solve the MOOPF problem with renewable sources. Section "Experimental results and analysis" presents the results of the experimental cases, which are then analyzed and discussed in depth. Finally, Section "Conclusion and future work" summarizes and discusses future work.

## Related work

### Traditional algorithms for solving the OPF problem

Since the OPF problem was raised^7^, many researchers have studied the OPF problem, and traditional methods for solving it include the Newton method^8^, quadratic programming^9^, linear programming^10^, and interior point method^11^. These methods mentioned in the previous question were used to solve the Optimal Power Flow problem in the early days, but these methods can only be solved for linear objective functions, which in turn can lead to an increase in error. In considering non-convex fuel costs with threshold effects, these methods will not be solved efficiently.

### Metaheuristic algorithms methods for solving the OPF problem

Researchers now have new solution ideas thanks to the emergence of metaheuristic algorithms, which have the benefit of not requiring them to concentrate on the objective function and constraints. Metaheuristic algorithms are widely used in solving OPF problems. In^12^ a new genetic algorithm for coding systems was applied to the OPF problem, the objective was to minimize fuel costs. The particle swarm optimization (PSO)^13^ algorithm was used to solve the OPF problem and test it on the IEEE 30-bus. In^14^, three new particle swarm optimization algorithms were used to find the optimal steady-state performance of power systems. Reference^15^ provided a new initialization method for the problem of genetic algorithms that may be ineffective if starting values of voltage angles are selected quite randomly. Mahadevan et al.^16^ applied a method named comprehensive learning particle swarm optimization (CLPSO) to the OPF problem with active power losses as objective functions. A new hybrid algorithm is proposed in^17^ optimal reactive power dispatch problem with discrete and continuous control variables. In^18^, a Gaussian bare-bones water cycle algorithm (NGBWCA) was proposed to minimize resistive losses and voltage deviations. In^19^, authors solved the Optimal Power Flow problem using a modified Sine–Cosine algorithm, this algorithm obtains a lower value of fuel cost and power losses. In^20^, Wei and Zhou et al. employed an improved slime mold algorithm (ISMA) to find optimal control parameters in power systems, and its effectiveness and robustness were also demonstrated. Recent literature^1^ proposed a high performance crisscross search based grey wolf optimizer (CS-GWO) to solve the OPF problem, fuel cost with valve-point effects and basic fuel cost are considered separately. Although the above study achieved the expected economic benefits, many researchers are not satisfied with achieving one optimization objective, and many researchers have also investigated the OPF problem with multiple optimization objectives. In^21^, multi-objective adaptive immune algorithm (MOAIA) was proposed for optimal reactive power flow incorporating static voltage stability. Reference^22^ provided a new variant of the differential evolutionary algorithm, ensures high convergence speed and diversity of Pareto solutions, and extracts the best compromise based on fuzzy set theory. Pulluri et al.^23^ proposed ESDE-MC methods to solve multi-objective OPF problem, non-dominated sorting and crowding distance was used in this method, the objectives to be optimized for the conflict include fuel costs, emissions, L-index and power losses. An improved NSGA-III (I-NSGA-III) was developed in^24^ to solve multi-objective OPF problems, the optimization goal is to simultaneously minimize total fuel cost, total emissions, voltage magnitude deviation and power loss. Although many classical meta-heuristic algorithms have been applied to solve this problem, many excellent algorithms have been proposed in recent years, such as the cheetah optimizer^25^, etc. Therefore, the use of novel algorithms to effectively improve the accuracy of problem solving is still worth investigating. In recently published literature^26,27^, the improved heap optimization algorithm and multi-objective Manta Ray Foraging Optimization were developed separately based on the Pareto concept, these two algorithms aim to simultaneously optimize four metrics: fuel cost, emissions, power loss and voltage deviation.

A summary of past research shows that many researchers have conducted exhaustive studies on single-objective and multi-objective problems for the OPF problem, and have obtained good results, but power systems are becoming more complex with the grid integration of renewable energy sources. Renewable energy sources have an element of uncertainty, yet the non-polluting nature of these sources compels us to use them, therefore, it is increasingly important to study the OPF problem with renewable energy. In^28^, the authors used the Weibull distribution to simulate the variability of wind, and then proposed a modified bacterial foraging algorithm to solve the OPF problem. On the basis of integrating wind power into the power grid^29^, also adding solar energy to the power grid, Lognormal probability distribution functions were used for forecasting solar photovoltaic power output. Reference^30^ considers the OPF problem with or without wind power and tested it on IEEE 30-bus, IEEE 57-bus and IEEE 118-bus respectively. In^31^, a novel hybrid modified imperialist competitive algorithm and sequential quadratic programming were proposed to solve the OPF problem, which studied the uncertainties of solar power and wind energy. Li and Gong^32^ proposed an enhanced adaptive different evolution and then applied it to the OPF problem on a modified IEEE 30-bus system, in which wind power and photovoltaic power are also being considered as energy supplies. A new version of the JAYA algorithm was proposed^33^ to solve the problem of OPF incorporating renewable energy sources, using a linear weighting method to integrating a multi objective OPF problem with four different objective functions into a single objective optimization OPF problem. In the most recent literature^34^, Li and Gong formulate the optimal power flow with stochastic wind and solar energy as a multi-objective optimization problem and a multi-objective evolutionary algorithm based on non-dominated sorting with constraint handling technique is presented to solve it. In^4^, tidal energy is also added as a new renewable energy source in the optimization of multi objective OPF problems. In^35,36^, novel heuristic algorithms Slime Mould Algorithm and Hunger Games Algorithm were used to solve single and multi-objective optimal power flow problems. The summary of related studies is listed in Table 1. Although this literature has studied OPF problems, including renewable energy, it is only the initial stage of research on OPF including renewable energy. This is because most studies in the literature only study the OPF problem with a single optimization objective in the grid with renewable energy supply, or use the linear weighting method to integrate multiple objectives into one optimization objective. It's important to reduce emissions and other indicators while reducing costs to the economy. Therefore, it can be concluded that the multi-objective OPF problem with renewable energy is worthy of further study^37,38^.

**Table 1:** Comparative review of different optimization algorithms in OPF problems.

References	Methodology	Test systems	Minimization Goals	Existence of renewable energy
^12^	Improved Genetic Algorithm (IGA)	IEEE 30-bus	Single objective: fuel costs	No
^13^	Particle swarm optimization	IEEE 30-bus	Single objective: fuel costs	No
^14^	Enhanced particle swarm optimization	IEEE 30-bus IEEE 118-bus	Single objective: power loss, voltage deviation	No
^15^	Genetic algorithm (GA)	IEEE 30-bus IEEE 118-bus	Single objective: fuel costs	No
^16^	Comprehensive learning particle swarm optimization (CLPSO)	IEEE 30-bus IEEE 118-bus	Single objective: power loss, voltage deviation	No
^17^	MICA-IWO	IEEE 30-bus IEEE 57-bus IEEE 118-bus	Single objective: power loss	No
^28^	Modified bacteria foraging algorithm	IEEE 30-bus	Single objective: fuel costs, voltage deviation, power loss	Wind power
^18^	Gaussian bare-bones water cycle algorithm	IEEE 30-bus IEEE 57-bus IEEE 118-bus	Single objective: voltage deviations	No
^28^	SHADE	IEEE 30-bus	Single objective: fuel costs, voltage deviation, power loss	Wind power and solar power
^19^	Sine–Cosine algorithm (SCA)	IEEE 30-bus IEEE 118-bus	Single objective: fuel costs, voltage deviation, power loss	No
^30^	Moth Swarm Algorithm (MSA)	IEEE 30-bus IEEE 57-bus IEEE 118-bus	Single objective: fuel costs, power loss	Wind power
^31^	Hybrid modified imperialist competitive algorithm	IEEE 30-bus IEEE 57-bus IEEE 118-bus	Single objective: fuel costs, emission	Wind power and solar power
^32^	Improved adaptive differential evolution (IADE)	IEEE 30-bus	Single objective: fuel costs, voltage deviation, power loss, emission	Wind power and solar power
^20^	Improved slime mold algorithm (ISMA)	IEEE 57-bus IEEE 118-bus IEEE 300-bus	Single objective: power loss	No
^1^	CS-GWO	IEEE 30-bus IEEE 118-bus	Single objective: fuel costs, voltage deviation, power loss	No
^21^	Differential evolution (DE)	IEEE 30-bus	Multi objectives: voltage deviation, power loss and voltage stability margin	No
^22^	MO-DEA	IEEE 30-bus IEEE 58-bus	Multi objectives: fuel costs, power loss and voltage profile improvement	No
^23^	ESDE-MC	IEEE 30-bus IEEE 58-bus Algerian 59-bus	Multi objectives: fuel costs, emission and power loss	No
^33^	MJAYA	IEEE 30-bus IEEE 118-bus	Multi objectives: fuel costs, emission and power loss	Yes
^24^	I-NSGA-III	IEEE 30-bus IEEE 57-bus IEEE 118-bus	Multi objectives: fuel costs, emission and power loss	No
^26^	Heap optimization algorithm	IEEE 57-bus IEEE 118-bus	Multi objectives: fuel costs, emission and power loss	No
^27^	IMOMRFO	IEEE 30-bus IEEE 57-bus	Multi objectives: fuel costs, emission and power loss	No
^34^	ACNSDE	IEEE 30-bus IEEE 57-bus	Multi objectives: fuel costs, emission and power loss	Wind power and solar power
^4^	MO-ACOPF	IEEE 30-bus	Multi objectives: fuel costs, power loss and voltage profile improvement	Wind power, solar power and tidal energy

## Mathematical models

The OPF problem can be regarded as an optimization problem, classical OPF problems are single-objective. In contrast, the multi-objective OPF problem has many advantages in its solution. It can achieve the optimization of multiple optimization goals in one solution, such as fuel cost emission, power loss and voltage deviation. Multi-objective is not only a solution, it is a solution set, which can give decision-makers more opportunities to choose a compromise solution. The mathematical model of the multi-objective OPF problem can be defined by the following:

1 $$Minimize{:} \;f(a,b) = \left\{ {f_{1} (a,b),\;f_{2} (a,b), \ldots ,\;f_{N} (a,b)} \right\}$$

2 $$\begin{array}{*{20}c} {s.t.} & {\quad g(a,b) \le 0} \\ {} & {\quad h(a,b) = 0} \\ \end{array}$$

where *N* is the number of objective functions, $f_{i}$ is the objective function to be optimized in a multi-objective OPF problem, *i* = 1,2,…,*N*; ***a*** and ***b*** are the control and state variable vectors, respectively. Multiple constraints need to be satisfied in the MOOPF problem, *g (a, b)* and* h (a, b)* represent the equality constraint and inequality constraint in the multi-objective OPF problem. The goal of solving the MOOPF problem is to find an optimal control variable ***a***, which minimizes fuel cost, emission, power loss and voltage deviation. The Control variable vector is given in (3).

3 $$a = [P_{{TG_{2} }} , \ldots ,P_{{TG_{NG} }} ,V_{{TG_{1} }} , \ldots ,V_{{TG_{NG} }} ,Q_{{SH_{1} }} , \ldots ,Q_{{SH_{NC} }} ]$$

where $P_{TG}$ is the active power of the thermal generators; $V_{{TG_{{}} }}$ is the voltage value of all generator unit buses; $Q_{{SH_{{}} }}$ is described as the shunt VAR compensation. *NG* is identified as the number of thermal generator buses in the test network, and NC is identified as the number of shunt compensators in the test network.

The state variable vector is given in (4)

4 $$b = [P_{{TG_{1} }} ,V_{{m_{1} }} , \ldots ,V_{{m_{NL} }} ,Q_{{TG_{1} }} , \ldots ,Q_{{TG_{NG} }} ,D_{{L_{1} }} , \ldots ,D_{{L_{nl} }} ]$$

where $P_{{TG_{1} }}$ represents the swing slack generating unit, $V_{{m_{r} }}$ indicates the voltage magnitude at the *r-th* load bus, *NL* is the number’s value of load buses; $Q_{{TG_{i} }}$ is regarded as the reactive power outputs at the *i-th* generator bus, $D_{{L_{{}} }}$ is the apparent power of the transmission lines, *nl* is the number’s value of the transmission lines.

### Constraints

The power system in the MOOPF problem needs to satisfy many power flow constraints, including equality constraints and inequality constraints.

#### Equality constraints

The equality constraint stems primarily from the reality that the generator's active power must equal the active load demand and active power loss. The equality constraints of MOOPF problem can be defined as:

5 $$P_{{TG_{i} }} = P_{{D_{i} }} + V_{i} \sum\limits_{j = 1}^{ND} {V_{j} \left[ {G_{ij} \cos (\theta_{i} - \theta_{j} ) + H_{ij} \sin (\theta_{i} - \theta_{j} )} \right]}$$

6 $$Q_{{TG_{i} }} = Q_{{D_{i} }} + V_{i} \sum\limits_{j = 1}^{ND} {V_{j} \left[ {G_{ij} \cos (\theta_{i} - \theta_{j} ) - H_{ij} \sin (\theta_{i} - \theta_{j} )} \right]}$$

where $P_{{D_{i} }}$ and $Q_{{D_{i} }}$ represent the active powers and the reactive load demands of the i-th load buses. $\theta_{i}$ define the i-th bus voltage angle. $G_{ij}$ and $H_{ij}$ are identified as the conductance and susceptance values of the transmission line between the *i-th* and *j-th* buses. *ND* is the number of buses, and *i* belong to bus number *1* to *ND*.

#### Inequality constraints

The inequality constraints of the MOOPF problem are described as:

(a) Generator constraints:

$$\begin{array}{*{20}c} {P_{{TG_{i} }}^{\min } \le P_{{TG_{i} }}^{{}} \le P_{{TG_{i} }}^{\max } } & {} \\ {Q_{{TG_{i} }}^{\min } \le Q_{{TG_{i} }}^{{}} \le Q_{{TG_{i} }}^{\max } } & {\quad \forall i \in NG} \\ {V_{{TG_{i} }}^{\min } \le V_{{TG_{i} }}^{{}} \le V_{{TG_{i} }}^{\max } } & {} \\ \end{array}$$

(b) Shunt compensator constraints:

$$Q_{{SH_{j} }}^{\min } \le Q_{{SH_{j} }}^{{}} \le Q_{{SH_{j} }}^{\max } \quad \forall j \in NC$$

(c) Transformer constraints:

$$T_{{K_{{}} }}^{\min } \le T_{K}^{{}} \le T_{{K_{{}} }}^{\max } \quad \forall K \in NT$$

(d) Contingency constraints:

$$V_{{m_{r} }}^{\min } \le V_{{m_{r} }}^{{}} \le V_{{m_{r} }}^{\max } \quad \forall r \in NL$$

$$D_{{L_{n} }}^{\min } \le D_{{L_{n} }}^{{}} \le D_{{L_{n} }}^{\max } \quad \forall n \in nl$$

where $T_{K}^{{}}$ regard as the k-th branch transformer tap. $P_{{TG_{i} }}^{{}}$, $Q_{{TG_{i} }}$, $V_{{TG_{i} }}$, $Q_{{SH_{j} }}$, $T_{K}^{{}}$, $V_{{m_{r} }}$ and $D_{{L_{n} }}^{{}}$ must limit between its upper ($P_{{TG_{i} }}^{\max }$, $Q_{{TG_{i} }}^{\max }$, $V_{{TG_{i} }}^{\max }$, $Q_{{SH_{j} }}^{\max }$, $T_{K}^{\max }$, $V_{{m_{r} }}^{\max }$, $D_{{L_{n} }}^{\max }$) and lower bounds ($P_{{TG_{i} }}^{\min }$, $Q_{{TG_{i} }}^{\min }$, $V_{{TG_{i} }}^{\min }$, $Q_{{SH_{j} }}^{\min }$, $T_{K}^{\min }$, $V_{{m_{r} }}^{\min }$, $D_{{L_{n} }}^{\min }$).

### The cost of energy spent in the system

#### Part of thermal generator set

Considering the valve point loading effect, the total cost of thermal power units ($C_{TG}$) is calculated as follows:

7 $$C_{TG} = \sum\limits_{i = 1}^{NG} {a_{i} + b_{i} P_{{TG_{i} }} + c_{i} } P_{{TG_{i} }}^{2} + \left| {d_{i} \cdot \sin (e_{i} \cdot (P_{{TG_{i} }}^{\min } - P_{{TG_{i} }} ))} \right|$$

where $C_{TG}$ regard as the thermal generation cost, $a_{i} ,\;b_{i} ,\;c_{i}$ are the cost calculation coefficients generated by the i-th thermal generator set, $d_{i} ,\;e_{i}$ are the cost calculation coefficients of the *i-th* thermal generator set due to the valve point loading effect. The specific values of these parameters will be given in the experiment in^34^.

#### Part of direct cost of wind, PV and tidal

Wind, photovoltaic and tidal power generation require the purchase and installation of equipment, so operators must pay this cost, which becomes the direct cost of renewable energy. These costs are proportional to the power of the equipment and can be calculated from (8), (9)^29^ and (10)^39^.

8 $$C_{Wd} = \sum\limits_{i = 1}^{Nw} {\alpha_{i} \cdot P_{ws,i} }$$

9 $$C_{Sd} = \sum\limits_{j = 1}^{Ns} {\beta_{j} \cdot P_{pvs,j} }$$

10 $$C_{Td} = \sum\limits_{l = 1}^{Nt} {\lambda_{l} \cdot P_{ts,l} }$$

where $\alpha_{i}$, $\beta_{j}$ and $\lambda_{l}$ represents the purchase and installation cost coefficient of the *i-th* wind power turbine, the *j-th* Solar photovoltaic panel and the *l-th* tidal power plant. $P_{ws,i}$, $P_{pvs,j}$ and $P_{ts,l}$ regard as the scheduled power of the *i-th* wind power farm, the *j-th* photovoltaic power plant and the *l-th* tidal power plant.

#### Part of uncertainty cost of wind, PV and tidal

Due to the uncertainty and intermittency of wind power, photovoltaic power generation and tidal energy, there will be two situations in which the demand for electricity is greater than the supply of renewable energy and the demand for electricity is less than the supply of renewable energy. When the first situation occurs, the power dispatching system needs to raise other thermal generating units to supplement the power supply to reach a state of equilibrium, so this situation will generate additional supplementary generation costs. In the second case, there will be a surplus of electricity generated from renewable sources, so the power dispatch system will have to pay for the extra renewable power^29^. The expenses paid by operators in these two instances are reserve expenses and penalty expenses, respectively. Equation (11) can be used to determine the uncertainty cost of wind power^29^.

11 $$\begin{aligned} C_{Wc} & = C_{Rw} + C_{Pw} \\ & = \sum\limits_{i = 1}^{Nw} {K_{Rw,i} \int_{0}^{{P_{ws,i} }} {(P_{ws,i} - P_{w,i} )f_{w} (P_{w,i} )dP_{w,i} } } \\ & \quad + \sum\limits_{i = 1}^{Nw} {K_{Pw,i} \int_{{P_{ws,i} }}^{{P_{wr,i} }} {(P_{w,i} - P_{ws,i} )f_{w} (P_{w,i} )dP_{w,i} } } \\ \end{aligned}$$

where $C_{Wc}$ is the total cost of wind energy source unknown. $C_{Rw}$ and $C_{pw}$ denotes the wind turbine's reserve and penalty costs, respectively. $K_{Rw,i}$ and $K_{Pw,i}$ indicate the i-th wind turbine's reserve and penalty cost coefficient, respectively. *Nw* denotes the total quantity of wind turbines in the power system. $P_{wr,i}$ is the rated output capacity of the i-th wind farm plant. $P_{ws,i}$ is defined as the planned out power from i-th wind farm plant. $P_{w,i}$ is the output capacity of the i-th wind farm plant.

Uncertainty cost of PV power can be calculated by (12)^29^.

12 $$\begin{aligned} C_{Sc} & = C_{Rs} + C_{Ps} \\ & = K_{Rs,i} \cdot f_{s} (P_{s,i} > P_{pvs,i} ) \cdot \left[ {E(P_{s,i} > P_{pvs,i} ) - P_{pvs,i} } \right] \\ & \quad + K_{Ps,i} \cdot f_{s} (P_{s,i} < P_{pvs,i} ) \cdot \left[ {P_{pvs,i} - E(P_{s,i} < P_{pvs,i} )} \right] \\ \end{aligned}$$

where $C_{Sc}$ is the total cost of solar electricity supply uncertainty. $C_{Rs}$ and $C_{Ps}$ represents the reserve and penalty expenses of the solar energy, respectively. $K_{Rs}$ and $K_{Ps}$ represent the reserve and penalty cost coefficient of solar energy source, respectively. $P_{s,i}$ is represent the actual generating capacity of the i-th solar power plant. $f_{s} (P_{s,i} > P_{pvs,i} )$ and $f_{s} (P_{s,i} < P_{pvs,i} )$ are defined as the probability of actual generating capacity more and less than the scheduled power, respectively. $E(P_{s,i} > P_{pvs,i} )$ and $E(P_{s,i} < P_{pvs,i} )$ represent the PV power expectancy above and below the scheduled power of the i-th PV power plant, correspondingly.

Uncertainty cost of tide power can be calculated by(13)^4,39^.

13 $$\begin{aligned} C_{Tc} & = C_{Rt} + C_{Pt} \\ & = K_{Rt} \cdot f_{t} (P_{t,i} > P_{ts} ) \cdot \left[ {E(P_{t,i} > P_{ts} ) - P_{ts} } \right] \\ & \quad + K_{Pt} \cdot f_{t} (P_{t,i} < P_{ts} ) \cdot \left[ {P_{ts} - E(P_{t,i} < P_{ts} )} \right] \\ \end{aligned}$$

where $C_{Tc}$ is the total cost of tidal electricity supply uncertainty.$C_{Rt}$ and $C_{Pt}$ represents the reserve and penalty expenses of the tidal energy. $K_{Rt}$ and $K_{Pt}$ represent the reserve and penalty cost coefficient of tidal energy source. $P_{t,i}$ represent the actual generating capacity of the *i-th* tidal power plant. $f_{t} (P_{t} > P_{ts} )$ and $f_{t} (P_{t} < P_{ts} )$ are defined as the likelihood of actual generating capacity being greater than or less than the planned tidal power plant capacity, respectively. $(P_{t,i} > P_{ts} )$ and $E(P_{t,i} < P_{ts} )$ are the expectancy of tidal power plant above and below the scheduled power of *i-th* tidal power plant, respectively.

### Objective function

The optimization of the MOOPF problem primarily includes economic and environmental optimization, and this paper employs four objective functions to measure and optimize economic and environmental indicators. The four objective functions are total generator cost, emission, power loss and voltage deviation. The total generators cost is defined as follows:

14 $$F_{1} = C_{TG} + C_{Wd} + C_{Sd} + C_{Td} + C_{Wc} + C_{Sc} + C_{Tc}$$

In order to promote the use of green energy, regulators need to assess the pollution generated by thermal generators. Therefore, the second target to be optimized is the emissions of thermal units in the power grid, which can be evaluated by (15).

15 $$F_{2} = \sum\limits_{i = 1}^{NG} {\left[ {(m_{i} + n_{i} \cdot P_{{TG_{i} }} + w_{i} \cdot P_{{TG_{i} }}^{2} ) \cdot 10^{ - 2} + t_{i} \cdot \exp (r_{i} \cdot P_{{TG_{i} }} )} \right]}$$

where, $m_{i} , \;n_{i} , \;w_{i} , \; t_{i}$ and $r_{i}$ represents some emission coefficients in the i-th thermal generator.

Reducing the active power loss in the line is also an important optimization objective. The third objective to be optimized is the active power loss, which can be calculated as follows:

16 $$F_{3} = P_{loss} = \sum\limits_{i = 1}^{nl} {\sum\limits_{j \ne i}^{nl} {G_{ij} \cdot \left[ {V_{i}^{2} + V_{j}^{2} - 2V_{i} V_{j} \cos (\theta_{i} - \theta_{j} )} \right]} }$$

Voltage deviation has a great influence on the voltage quality in the power system, so voltage deviation will be the fourth target to be optimized. Voltage deviation in the MOOPF problem can be calculated as follows:

17 $$F_{4} = VD = \sum\limits_{r = 1}^{NL} {\left| {V_{{m_{r} }} - 1.0} \right|}$$

**Figure 1:**
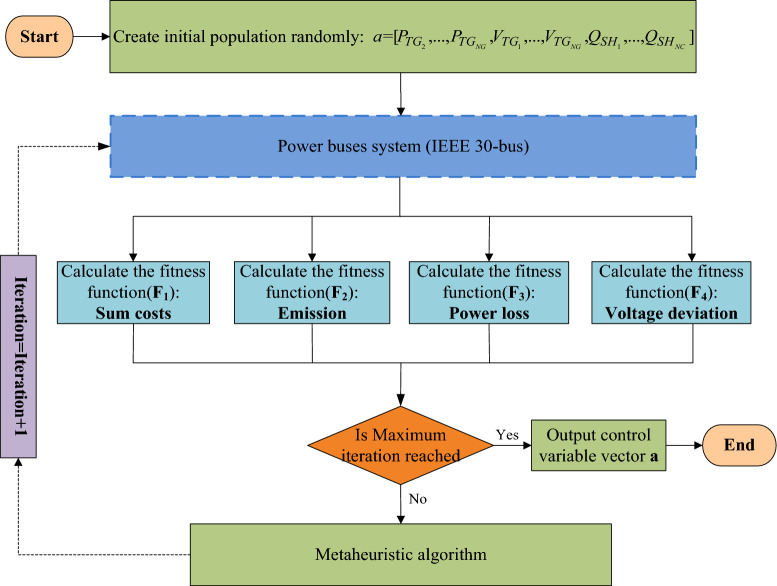
A framework for solving MOOPF problem by metaheuristic algorithm.

According to the description of the model, to solve the MOOPF problem, we need to search for a control variable vector ***a***, and the make the four objective functions minimized at the same time. In addition, ***a*** needs to satisfy the constraints. Because solving the MOOPF problem with renewable energy is extremely difficult, we suggest a general framework for solving it. The general framework for solving the MOOPF problem using metaheuristics is shown in Fig. 1.

## Uncertainty and power models for renewable energy

The stochastic modeling of renewable energy in the model will be described in this section.

### Wind speed, solar radiation and tidal flow probability

Wind speed is characterized by intermittent and uncertainty. It is well known that probability density functions can be used to calculate the mean output of wind turbines^28–30^. The wind speed (v) m/s follows a Weibull probability distribution function and is calculated using the scale parameter ($\gamma$) and form parameter ($\phi$) as shown below. Many researchers have studied the probability distribution of wind speed, and the Weibull distribution^28^ is considered to be a probability model that can well fit the distribution of wind speed. Wind speed likelihood can be determined using the Weibull probability density function as follows:

18 $$f_{v} (v) = \left( {\frac{\phi }{\gamma }} \right) \cdot \left( {\frac{v}{\gamma }} \right)^{\phi - 1} \cdot \exp \left( { - \left( {\frac{v}{\gamma }} \right)^{\phi } } \right)$$

where *v* is defined as the wind speed, $\phi$ and $\gamma$ are represents the shape and scale parameters. With the values provided in Table 5, $\phi$ and $\gamma$ were carefully selected to ensure both diversity and realistic geographic locations for wind farm sites.

The study discovered that where meteorological conditions are more dispersed, the lognormal function accurately represents the frequency distribution^40^. Use the lognormal probability density function to model solar irradiance^40^ for the solar probability, which can be described as follows:

19 $$f_{s} (S) = \frac{1}{{S \cdot \delta \cdot \sqrt {2\pi } }}\exp \left( {\frac{{ - (\log_{10} S - \mu )^{2} }}{{2\delta^{2} }}} \right)\quad S > 0$$

where *S* represents the solar irradiance, $\delta$ and $\mu$ are equal to 0.6 and 6, respectively.

In earlier works, Gumbel distribution was typically used to calculate the probability model of flow rate in the tidal range^4,39^. Equation (20) is calculates the likelihood of tidal energy transfer.

20 $$f_{t} (Q) = \frac{1}{\lambda } \cdot \exp \left( {\frac{Q - \varphi }{\lambda }} \right) \cdot \exp \left( { - \exp \left( {\frac{Q - \varphi }{\lambda }} \right)} \right)$$

From the result reported in^33^, the IEEE 30-bus system was modified to include the supply of wind energy, solar energy and tidal energy. In order to show the comparability of the results, the parameters of the probability distribution function from the previous literature are used in this paper. Different parameters can enhance the diversity and uncertainty of the renewable energy supply. The PDF parameters of these renewable energy sources are detailed in section "Result on the modified IEEE 30-bus with renewable energy".

### Wind turbine and solar panel power model

Wind turbines and solar panels will provide electricity for the electrical network. Therefore, their power output needs to be calculated according to the wind speed probability and solar radiation probability in the previous section. The turbine actual output power is a function of wind speed^28^, the function can be formulated as follows:

21 $$P_{w} (v) = \left\{ {\begin{array}{*{20}c} 0 & {\quad v_{out} < v\;or\;v < v_{in} } \\ {P_{wr} \cdot \left( {\frac{{v - v_{in} }}{{v_{r} - v_{in} }}} \right)} & {\quad v_{in} \le v \le v_{r} } \\ {P_{wr} } & {\quad v_{r} < v \le v_{out} } \\ \end{array} } \right.$$

where $v_{in}$ = 3 m/s,$v_{out}$ = 25 m/s are defined as the cut-in and cut-out wind speeds, $v_{r}$ = 16 m/s represents the rated wind speed, $P_{wr}$ = 3 MW is the wind turbine rated output power. The turbine has three states in wind speed. In the first situation, v < $v_{in}$ or v > $v_{out}$, the wind turbine will be stationary or locked to protect the speed does not exceed the limit rotor speed. In the second situation, $v_{in} \le v \le v_{r}$, the wind turbine will output power, according to wind speed. In the finally situation, $v_{r} < v \le v_{out}$, the wind turbine will be continuing to produce electricity at rated power. According to, the probabilities of three different cases can be calculated as follows:

22 $$f_{w} \left\{ {P_{w} = 0} \right\} = 1 - e^{{ - \left( {\frac{{v_{in} }}{\omega }} \right)^{k} }} + e^{{ - \left( {\frac{{v_{out} }}{\omega }} \right)^{k} }}$$

23 $$f_{w} \left\{ {P_{w} = P_{wr} } \right\} = e^{{ - \left( {\frac{{v_{r} }}{\omega }} \right)^{k} }} - e^{{ - \left( {\frac{{v_{out} }}{\omega }} \right)^{k} }}$$

24 $$\begin{aligned} f_{w} \left\{ {P_{w} } \right\} & = \frac{{k(v_{r} - v_{in} )}}{{\omega^{k} \cdot P_{wr} }} \times \left[ {v_{in} + \frac{{P_{w} }}{{P_{wr} }}(v_{r} - v_{in} )} \right]^{k - 1} \\ & \quad \times \;\exp \left[ { - \left( {\frac{{v_{in} + \frac{{P_{w} }}{{P_{wr} }}(v_{r} - v_{in} )}}{\omega }} \right)^{k} } \right] \\ \end{aligned}$$

According to, the solar panel’s electricity output is a function of solar irradiance (*S*), which is defined as follows^40^:

25 $$P_{s} (S) = \left\{ {\begin{array}{*{20}c} {P_{sr} \left( {\frac{{S^{2} }}{{S_{std} \cdot R_{c} }}} \right)} & {\quad 0 < S < R_{c} } \\ {P_{sr} \left( {\frac{{S^{{}} }}{{S_{std} }}} \right)} & {\quad S \ge R_{c} } \\ \end{array} } \right.$$

where, $P_{sr}$ is the rated output power of the solar panel unit. $S_{std}$ = 800 W/m^2^ is the standard environment’s solar irradiance.$R_{c}$ = 120 W/m^2^ is a certain irradiance point.

**Figure 2:**
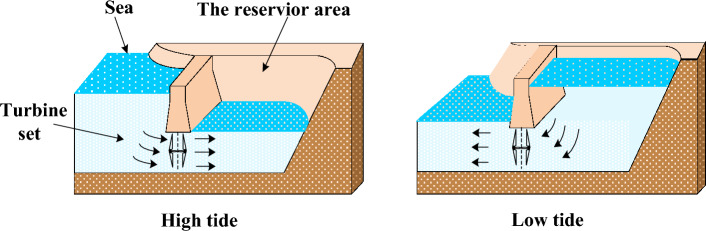
Tidal power generation process.

Tidal power, which generates electricity, was used by the sea water enters the reservoir at high tide to turn turbines, and then retreats from the reservoir back to the sea at low tide to turn turbines. Figure 2 shows the process of using tidal energy to generate electricity during high and low tides. The generating capacity of turbines in a tidal power plant can be calculated by (26)^39^.

26 $$P_{t} (Q) = \rho g Q H \varepsilon$$

where $\rho$ is the water density (kg/m^3^), $g$ is the gravity acceleration (m/s^2^), *Q* is the discharge value (m^3^/s) across the turbine set. $\varepsilon$ is the turbine efficiency, *H* is the difference in height between the reservoir and the sea surface. These parameters of the tidal system sere set as *H* = 3.2 m, $\rho$ = 1025 kg/m^3^, $\varepsilon$ = 0.85 and *g* = 9.81 m/s^2^.

## Multi objective pathfinder optimization algorithm (MOPFA)

The MOPFA will be suggested first in this part. Single Pathfinder Algorithm was proposed by Yapici and Cetinkaya in 2019, PFA has a strong global search capability, but the algorithm only deals with the single objective optimization problem. So in this research, we proposed the MOPFA, and then applied it to solve the MOOPF problem. Similar to the multi-objective particle swarm optimization algorithm (MOPSO)^41^. The first component is the archive, which is used to store the Pareto optimal solutions found so far. The pathfinder role selection process, which chooses the most likely pathfinder from the database and can guide the group to the optimum area, is the second component.

### Pareto dominance

When faced with solving a multi objective optimization problem^42^, $$x_{1}$$ and $$x_{2}$$ are two solutions, $$f_{i}$$ is the *i-th* objective function in this problem. If all the values of the objective function calculated by $$x_{1}$$ have the same or at least one better value than all the values of the objective function calculated by $$x_{2}$$, $$x_{1}$$ is said to dominate $$x_{2}$$_._ This relationship can be symbolically expressed as $$x$$ $$\succ$$$$y$$. If there is no $$x$$ in feasible solution set makes $$x$$$$\succ$$$$x^{^{\prime}}$$, $$x^{^{\prime}}$$ is defined as Pareto optimal solution. All the Pareto optimal solutions are combined into a set, which is Pareto optimal solution set. In addition, the set of values of the multiple objective function corresponding to the Pareto optimal solution set is called the Pareto front^42^.

### PFA population initialization

The MOPFA's first stage is to initialize the population so that it is evenly dispersed in the search area, which is accomplished using (27):

27 $$x = LB + rand(0,\;1) \cdot (UB - LB)$$

where, *x* is the position vector of the individual population, *LB* and *UB* are the upper and lower bounds of the problem.

### External archive initialization and update rules

The external repository holds the non-dominated optimal solutions as well as the collection of non-dominated optimal solutions found prior to the current run. The archive's capacity is fixed, typically half the size of the populace. The non-dominated optimal solutions derived from the initialize population were appended to the external archive component when it was started. The archive revised criteria can be specified as follows in subsequent iterations:

- Situation 1: If a new solution created as a result of a PFA update is dominated by at least one solution in the archive, the new solution was unable to join the archive.
- Situation 2: If a new solution after the PFA update dominated one or more of the solutions in the archive, the dominated solution is deleted and substituted with this new solution.
- Situation 3: If neither the new solution, nor archive members dominate each other, the new solution should be If neither the new solution nor the archive users outnumber each other, the new solution should be included in the archive.
- Situation 4: If a new solution after the PFA update dominates all the solutions in the archive, but the external cache is filled. In this scenario, according to the crowing distance indicator, a non-dominated answer in the archive will be eliminated.

In situation 4, crowding distance is a measure of the distance between one non-dominant solution and other adjacent non-dominant solutions in the archive. Equation (28) can be used to compute the crowding distance.

28 $$d = \frac{Max - Min}{{Archive\_size}}$$

where, *Max* and *Min* represent the maximum and minimum values of each objective, respectively. $Archive\_size$ is the archive’s capacity. According to (28), the crowing distance index *d*_*indicator*_ is defined as the number of neighboring solutions that are less than distance *d*. A roulette technique was used to arbitrarily delete a solution from the external archive in order to add a new non-dominated solution to the complete external archive. Assign a probability to each non-dominated solution in the archive according to the crowding distance indicator, this fitness calculation is completed by (29).

29 $$P_{i} = \frac{{d_{indicator} }}{{N_{sum} }}$$

where, $P_{i}$ is the probability of a non-dominant solution in an external archive is selected to be deleted by roulette method.$N_{sum}$ is the sum of the crowding distance index of each non-dominant solution in the external archive.

### Pathfinder individual update rules

A non-dominant solution set stored in an external archive can be regarded as an elite individual, in the pathfinder algorithm, the population was divided into followers and pathfinders, and pathfinders led the population to the most promising region. Because elite archiving and the pathfinder's leader behavior are both elite leader behaviors, treat the external archive as the pathfinder individual in the PFA and update it using the pathfinder's individual update rules in the PFA and the optimal protection strategy. The pathfinder updating position is obtained from (30).

30 $$x_{p}^{i + 1} = x_{p}^{i} + 2r_{3} \times (x_{p}^{i} - x_{p}^{i - 1} ) + \eta$$

where $x_{p}^{i + 1}$ indicates the place of the *i* + 1 generation pathfinder, $x_{p}^{i}$ is the location of the *i-th* pathfinder. $x_{p}^{i - 1}$ the *i *− 1 generation pathfinder's position, *i* represents the number of current iterations, $r_{3}$ represents a random integer from a uniform distribution at [0, 1]. $\eta$ is derived from (21).

31 $$\eta = u_{2} \cdot e^{{\frac{ - 2i}{{i_{\max } }}}}$$

where $i_{\max }$ indicates the highest number of iterations,$u_{2}$ is a random integer evenly spread between [− 1, 1].

### Follower individual update rules

The external archive stores the non-dominant solutions found so far, which can be regarded as people in the population's most hopeful area. The pathfinder individual description in Single objective PFA is matches. Different from PFA, in MOPFA treat population individuals as followers and external archives as pathfinders. According to the size of follower population, the corresponding pathfinder matrix is constructed, follower’s update process as follows:

32 $$x_{n}^{i + 1} = x_{n}^{i} + W_{1} \cdot (x_{n - 1}^{i} - x_{n}^{i} ) + W_{2} \cdot (x_{p}^{i} - x_{n}^{i} ) + \varepsilon$$

where *i* represent the current iteration, $x_{n}^{{}}$ represents the position of the follower of the population*.* $W_{1}$, $W_{2}$ are two randomly generated vectors calculated used Eqs. (33) and (34), $r_{1}$ and $r_{2}$ are a uniformly distributed random number generated randomly between [0, 1]. $W_{1}$,$W_{2}$ can control the weight of the follower moving to the pathfinder and the neighboring individuals in the population. $\varepsilon$ is the vibrancy vector, and its calculation can be obtained from (35), $\Delta_{ij}$ is the distance between the *i-th* and the *j-th* position in population.

33 $$W_{1} = \alpha \cdot r_{1}$$

34 $$W_{2} = \beta \cdot r_{2}$$

35 $$\varepsilon = \left( {1 - \frac{i}{{i_{\max } }}} \right) \cdot u_{1} \cdot \Delta_{ij} ,\Delta_{ij} = \left\| {x_{i} - x_{i - 1} } \right\|$$

**Figure 3:**
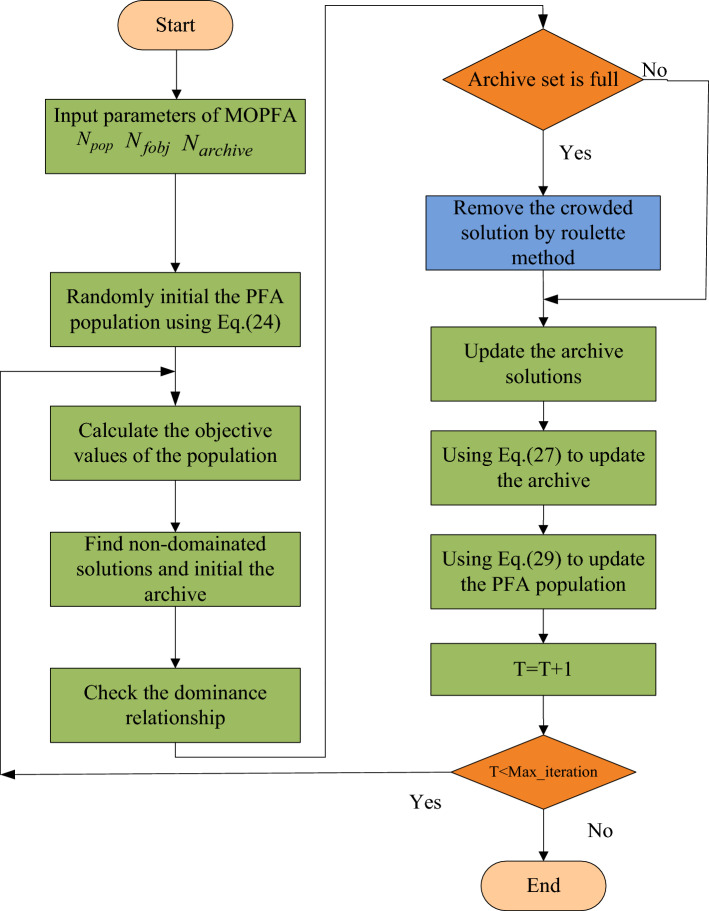
Flowchart of the multi-objective pathfinder algorithm (MOPFA).

The pseudo code of MOPFA is given in Algorithm 1, and the flowchart of the multi-objective pathfinder algorithm is shown in Fig. 3.

**Table:** 

**Algorithm 1.** Pseudo code of the multi-objective pathfinder algorithm (MOPFA)
1. Initialize parameters of MOPFA, *N*_pop_, *N*_fobj_, dim
2. Randomly generate the location of the search agent using (27)
3. **While** *i* < Max_iter
4. Calculate the fitness of each search agent
5. Find non-dominated solutions from the population
6. Check the dominance relationship between the non-dominant solutions in the current population and solutions in the archived
7. **If** archive is full
8. According to the crowing distance and roulette method remove solution in the archive
9. Update the archive
10. else
11. Update the archive
12. **End If**
13. Using (30) to update the archive
14. **If** new archive solution is better than old
15. Accept new archive solution
16. **End If**
17. Using (32) to update the follower population
18. **If** new follower population is better than old
19. Accept new population
20. **End If**
21. **End while**
22. **Output** the best optimal solutions

## Experimental results and analysis

In this part, MOPFA is used to answer the multi objective optimal power flow issue (MOOPF), and the experimental findings are thoroughly examined. Generation cost, power loss, voltage deviation and emission were studied as objective functions in this research. In order to study the emission reduction effect of renewable energy in the MOOPF problem, renewable energy is included in the energy source of the power network. The experiment was conducted on an adapted IEEE 30-bus. The power flow formulae of the suggested MOOPF model with renewable source were calculated using MATPOWER 6.0. Four cases were studied for different optimization purposes. Case 1 is to reduce both production costs and emissions at the same time. Case 2 and Case 3 are focused on three objectives. The most difficult is Case 4, which aims to optimize all four objective functions at the same time. The specific experiments case content is listed in Table 2. The optimization results of MOPFA and several other multi-objective optimization algorithms on modified IEEE 30-bus for MOOPF problem with four cases are listed in this section. The Hyper-Volume (HV) indicator^43^ obtained by MOPFA and other multi-objective optimization algorithms under four optimization objective cases were compared and also the results were also compared using statistical test analysis. Using the well-known fuzzy decision system^44^ to choose a consensus answer from a collection of Pareto optimum solutions. The experimental findings were then compared to newly published literature. Finally, the best intermediate solution's load bus voltage is validated. Each objective function for a non-dominant answer will initially be assigned a membership function value by the well-known fuzzy judgment system.

**Table 2:** Specific experiments content in different cases.

System	No.	Generation cost	Emission	Real power loss	Voltage deviation
IEEE 30-bus	Case 1	•	•		
	Case 2	•	•	•	
	Case 3	•	•		•
	Case 4	•	•	•	•

### Experimental setting

In order to better test the ability of MOPFA to solve the MOOPF problem, it is compared with six other excellent multi objective metaheuristic algorithms with the strong optimization ability, which are MOPSO^41^, NASGA-II^44^, MOSSA^45^, MOMVO^46^ and MOAHA^47^. The population, number of all metaheuristic algorithms $N_{pop}$ = 100 and the number of iterations $Maxiteration$ = 200. The outcomes of 30 runs are examined in the experimental verification to better attempt the algorithm's optimizing ability. Each metaheuristic algorithm's particular parameter values are consistent with the parameters of the original algorithm. The simulations were performed on the MATLAB 2016b platform and run on a CPU Core i5-7100 v5 (3.80 GHz) with 16 GB RAM.

### HV indicators

In the multi-objective optimization algorithm, the quality of the algorithm needs to be measured through a variety of indicators. The HV indicator is a comprehensive evaluation indicator, and can be compared without the real Pareto frontier. The higher the HV value is, the better the algorithm effect will be.

### Fuzzy decision system

In this paper, the following fuzzy decision system is used to select the compromise solution in the Pareto optimal solution set. The calculation method is as follows^44^:

36 $$\gamma_{n}^{k} = \left\{ {\begin{array}{*{20}c} 1 & {\quad f_{n}^{k} \le f_{n}^{\min } } \\ {\frac{{f_{n}^{\max } - f_{n}^{k} }}{{f_{n}^{\max } - f_{n}^{\min } }}} & {\quad f_{n}^{\min } \le f_{n}^{k} \le f_{n}^{\max } } \\ 0 & {\quad f_{n}^{k} \ge f_{n}^{\max } } \\ \end{array} } \right.$$

where $\gamma_{n}^{k}$ is the membership function value of *n-th* objective for k-th non-dominated solution; $f_{n}^{k}$ is the fitness value of *n-th* objective for the *k-th* non-dominated solution; $f_{n}^{\min }$ and $f_{n}^{\max }$ are the minimum and maximum fitness values for the *n-th* objective function among all non-dominated solutions. The normalized membership function for each non-dominated solution is defined as:

37 $$\gamma^{k} = \frac{{\sum\nolimits_{n = 1}^{N} {\kappa_{n}^{k} } }}{{\sum\nolimits_{k = 1}^{{N_{d} }} {\sum\nolimits_{n = 1}^{N} {\kappa_{n}^{k} } } }}$$

where, *N* is the number of objectives, for example *N* is 3 in Case 2, *N* is 3 in Case 2 and Case 3, *N* is 4 in Case 4. $N_{d}$ is the sum of non-dominated solutions. The best compromise solution is a solution in non-dominated solutions set with maximum $\gamma^{k}$ value.

### Result on the modified IEEE 30-bus with renewable energy

**Figure 4:**
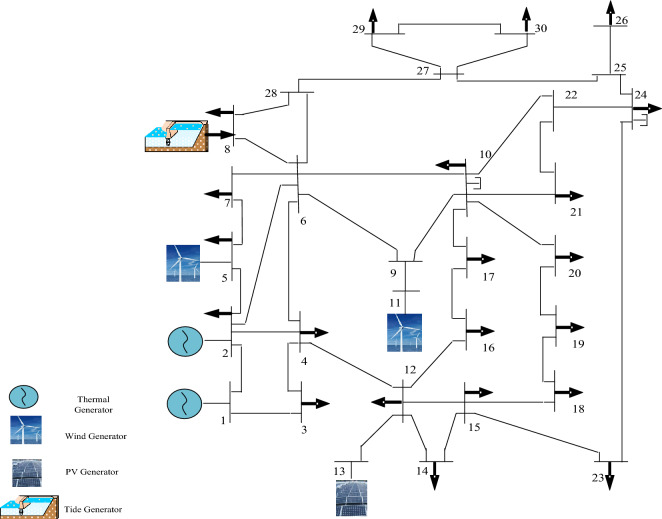
The Modified IEEE 30-bus.

To integrate renewable energy into the grid’s electricity supply, IEEE 30-bus was modified in this study. According to Ref.^28^, the system consisted of 41 transmission lines, 6 generating units, 9 shunt VAR compensators, and 4 transformer tap settings and its total active and reactive load demands were 283.4 MW and 126.2 MVAR, respectively. In the modified IEEE 30-bus the thermal generators at buses 5, 11 were replaced by wind generators respectively, wind generator farm at bus 5 has 15 turbines, and bus 11 has 10 turbines. The thermal generators at buses 13 and 8 were replaced by solar generator and tidal generators respectively, tidal generator at bus 8 has 4 generating sets. Table 3 describes the modified IEEE 30-bus settings, and the renewable uncertainty cost coefficients are listed in Table 4^29^. The modified IEEE 30-bus structure diagram was shown in Fig. 4. Parameter values of the probability distribution function of simulated renewable energy in all Cases 1–4 are given in Table 5.

**Table 3:** The modified IEEE 30-bus settings.

Items	Quantity	Description
Bus	30	
Branch	41	
Thermal generator	3	At buses 1, 2
Wind generator	2	At buses 5 and 11
Solar PV generator	1	At bus 13
Tidal generator	1	At bus 8
Control variable	11	Scheduled real power for 5 generators except bus 1; bus voltage of 6 generator buses
Connected load		283.4 MW 126.2 MVAr
Load bus voltage	24	Allowed range; [0.95–1.05] p.u

**Table 4:** Renewable energy cost coefficients.

Wind power farm ($/MW)				Wind power farm ($/MW)			
Bus no.	Direct cost	Reserve cost	Penalty cost	Bus No	Direct cost	Reserve cost	Penalty cost
5	1.60	3	1.5	11	1.75	3	1.5

Solar power plant ($/MW)				Tidal power plant ($/MW)			
Bus no.	Direct cost	Reserve cost	Penalty cost	Bus no.	Direct cost	Reserve cost	Penalty cost
13	1.60	3	1.5	8	3.0	3	1.5

**Table 5:** Renewable energy probability in modified IEEE 30-bus test system according to Eqs. (18–20).

Wind power plant (bus 5)			Wind power plant (bus 11)			Solar generators (bus 13)		Tidal power plant (bus 8)	
Number of turbines	Rated power	Weibull PDF parameters	Number of turbines	Rated power	Weibull PDF parameters	Rated power	Lognormal PDF parameters	Rated power	Gumbel PDF parameters
25	75 MW	K = 2, 1 = 10	20	60 MW	K = 2, λ = 9	50 MW	0.6 ,6	40 MW	220,24.52

#### Result on case 1: minimize the generator cost and emission

In this case, the goal of optimization is to minimize generation costs and emissions. Cost and emission are in conflict, but power system generation cost and emission control should also be given more attention to. After the 30 runs, the Hyper-Volume (HV) indicator obtained by MOPFA and other algorithms in Case 1 are listed in Table 6, which statistically analyzes the maximum (Max), average (Mean) and minimum (Min) values of each algorithm in the 30 runs and the best results are highlighted in boldface. Furthermore, in order to better test the performance of the algorithm, the Wilkerson rank sum test is used to rank the algorithm.

**Table 6:** HV-indicator in Case 1.

Case	Algorithm	HV					
		Max	Min	Mean	Std	Score	Rank
Case 1	MOPFA	**0.18025**	**0.14239**	**0.14773**	0.0064009	**4.87**	**1**
	NASGA-II	0.13307	0.02018	0.06352	0.030878	1.03	6
	MOPSO	0.16488	0.10165	0.13857	0.013743	3.57	4
	MOMVO	0.16829	0.12083	0.14649	0.0091062	4.55	2
	MOAHA	0.15845	0.13968	0.14647	**0.0047576**	4.53	3
	MOSSA	0.14884	0.086813	0.11812	0.017829	2.47	5

**Figure 5:**
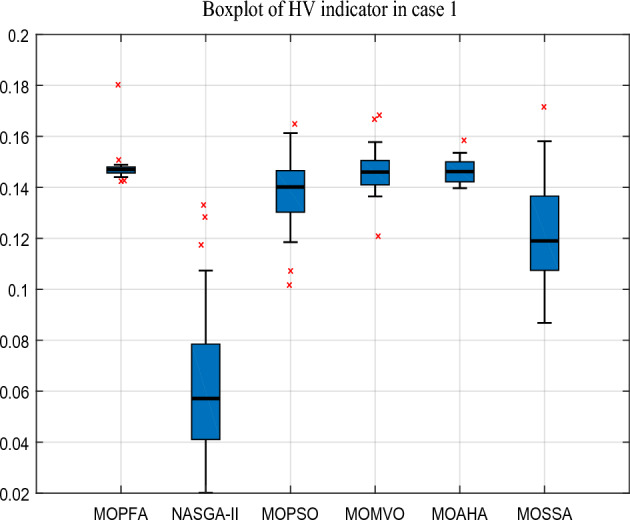
Boxplot of HV indicator in Case 1.

**Figure 6:**
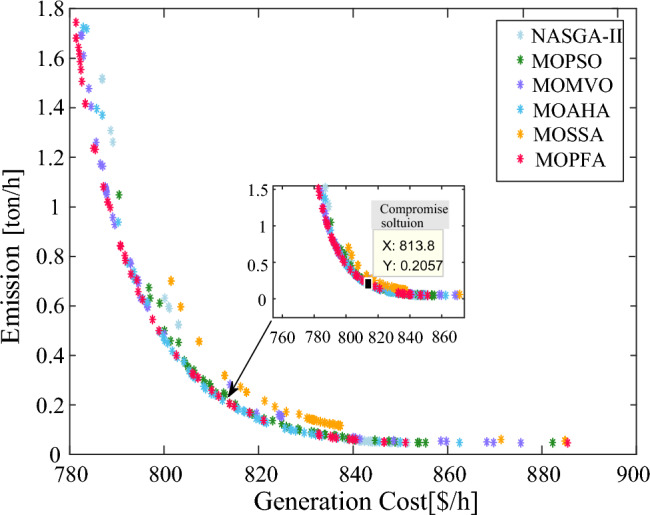
Pareto front in Case 1.

From Table 6, it can be calculated that MOPFA obtained the maximum HV indicator of **0.18025** and the minimum value of **0.1423** after 30 runs, which was better than other algorithms. The average value was also compared, MOPFA achieved **0.14773**, ranking first. Although the mean square deviation value was not as good as MOAHA's, the gap was also small. MOPFA's Wilkerson rank sum test score is **4.87**, ranking it first among the six multi-objective algorithms. The boxplot figure of the HV indicator obtained by each algorithm on Case 1 is given in Fig. 5. The Pareto front obtained by the HV index maximum runtime algorithm is plotted in Fig. 6. It can be seen that the Pareto front obtained by MOPFA is more uniform and MOPFA can give a more diverse set of Pareto optimal solutions.

**Figure 7:**
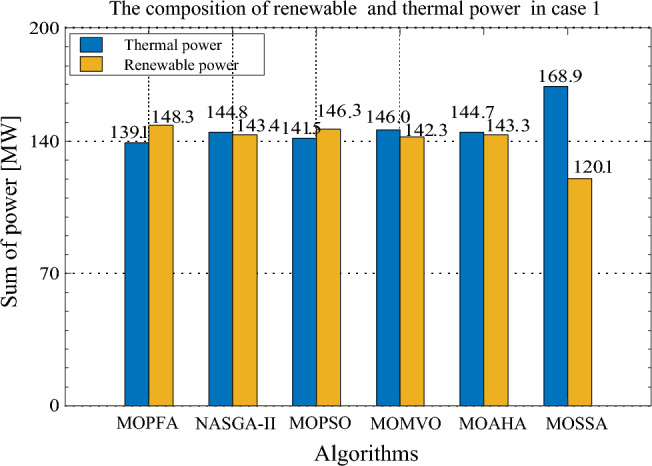
The composition of renewable and thermal power in Case 1.

According to the fuzzy decision system mentioned in the preceding part of this paper, the compromise solutions were selected from the Pareto front in Fig. 6. The compromise solutions on Case 1 of MOPFA and other well-known multi-objective optimization algorithms are reported in Table 7. The composition of renewable generation energy and thermal generation energy in the solution obtained by each algorithm is shown in Fig. 7. The compromise solution obtained by MOPFA is marked in Fig. 6, the value of generation cost is 813.8379 ($/h) and emission is 0.2057 (t/h).

**Table 7:** Compromise solution values obtained by each algorithm. Significant values are in bold.

	LB	UB	Algorithm					
			MOPFA	NASGA-II	MOPSO	MOMVO	MOAHA	MOSSA
Control variables								
$$P_{{G_{2} }} \;({\text{MW}})$$	20	80	40.4386	29.2474	40.2342	31.6149	37.4524	48.3333
$$P_{{G_{5} }} \;({\text{MW}})$$	0	40	23.0599	26.3456	23.7595	22.6247	22.0329	22.1281
$$P_{{G_{8} }} \;({\text{MW}})$$	0	75	46.9693	46.4971	46.9788	47.5637	44.7445	37.2967
$$P_{{G_{11} }} \;({\text{MW}})$$	0	60	37.1813	37.1798	38.1290	33.1001	37.2897	29.6634
$$P_{{G_{13} }} \;({\text{MW}})$$	0	50	41.1541	33.3938	37.5290	39.0482	39.2914	31.0788
$$V_{1} \;({\text{P.U.}})$$	0.95	1.10	1.0588	1.0617	1.0620	1.0762	1.0611	1.0715
$$V_{2} \;({\text{P.U.}})$$	0.95	1.10	1.0385	1.0487	1.0271	1.0661	0.9776	1.0379
$$V_{5} \;({\text{P.U.}})$$	0.95	1.10	1.0955	1.0505	1.0293	1.0902	1.0803	1.0356
$$V_{8} \;({\text{P.U.}})$$	0.95	1.10	1.0851	1.0857	1.0563	1.0941	1.0796	1.0994
$$V_{11} \;({\text{P.U.}})$$	0.95	1.10	1.0968	1.0350	1.0704	1.0381	1.0913	1.0946
$$V_{13} \;({\text{P.U.}})$$	0.95	1.10	1.0914	1.0850	1.0828	1.0013	1.0888	1.0992
State variables								
$$P_{{G_{1} }} \;({\text{MW}})$$			98.7111	115.6094	101.3245	114.4035	107.3417	120.6074
$$Q_{{G_{1} }} \;({\text{MVAr}})$$			3.3609	− 2.5389	19.0039	− 2.6376	6.7935	18.1485
$$Q_{{G_{2} }} \;({\text{MVAr}})$$			− 20	5.9618	− 20	27.2211	− 20	− 20
$$Q_{{G_{5} }} \;({\text{MVAr}})$$			35	35	35	40	35	35
$$Q_{{G_{8} }} \;({\text{MVAr}})$$			40	40	31.4838	40	35	29.9355
$$Q_{{G_{11} }} \;({\text{MVAr}})$$			29.8692	10.2685	23.1952	12.3685	27.3617	28.3307
$$Q_{{G_{13} }} \;({\text{MVAr}})$$			25	25	25	1.7404	25	25
Objectives								
F1: Generation Cost [$/h]			813.8379	803.0716	812.4313	**800.0093**	805.6819	801.4793
F2: Emission [ton/h]			**0.2057**	0.5224	0.2351	0.4864	0.3250	0.7013

From Table 7, it can be calculated that MOPFA obtained the best emission value: 0.2057, but MOMVO obtained the best generation cost value: 800.0093. From Fig. 7, it can be seen why MOPFA's solution did not achieve the best cost. Because MOPFA’s solution uses the total thermal power and renewable energy is 139.1494 MW ($P_{{G_{1} }}$ + $P_{{G_{2} }}$) and 148.3646 MW ($P_{{G_{8} }}$ to $P_{{G_{13} }}$), while MOMVO’s solution uses the total thermal power and renewable energy is 141.5587 MW ($P_{{G_{1} }}$ + $P_{{G_{2} }}$) and 143.4163 MW ($P_{{G_{8} }}$ to $P_{{G_{13} }}$). MOPFA’s solution uses more renewable energy and renewable energy uncertainty will increase the generation cost, But MOPFA's 0.2057 (ton/h) emissions are 50% lower than MOMVO's 0.4864 (ton/h). The total generated power of six algorithms are 287.4 MW, 288.2 MW, 287.8 MW, 288.3 MW, 288.0 MW and 289.0 MW respectively, which achieves the power system required load 286.949 MW shown in Table 3. Compare MOPFA’s compromise solution with the published literature was reported in Table 8, MOPFA’s generation cost is lower than other algorithms except ACNSDE-SF^30^, but MOPFA’s emission is lower than ACNSDE-SF. Both generation cost and emission are considered, MOPFA obtained value is lower than MODFA^32^, MOEA/D-SF^33^, ESDE^19^, PSO-SSO^34^ and MOMICA^35^. In summary, the solutions given by each algorithm are not dominated by each other, and the decision maker decides which one to choose, but MOPFA's HV index of the Pareto front is higher, indicating that the Pareto front is more evenly distributed, and it can provide a greater diversity of solutions. Moreover, MOPFA's compromise solution is more inclined toward the utilization of renewable energy, which will reduce the spending of enterprises when the government imposes higher emission taxes.

**Table 8:** The compromise solution of Case 1 obtained by MOPFA was compared with the published literature. Significant values are in bold.

Algorithm	Generation cost [$/h]	Emission [ton/h]
MOPFA	813.8379	**0.2057**
SHADE-SP^29^	**782.503**	1.762
ACNSDE-SF^34^	843	0.123
MODFA^48^	831.665	0.2432
MOEA/D-SF^49^	829.515	0.2501
ESDE^23^	833.474	0.2540
PSO-SSO^50^	834.804	0.243
MOMICA^51^	865.06	0.222

#### Result on Case 2 and Case 3

**Figure 8:**
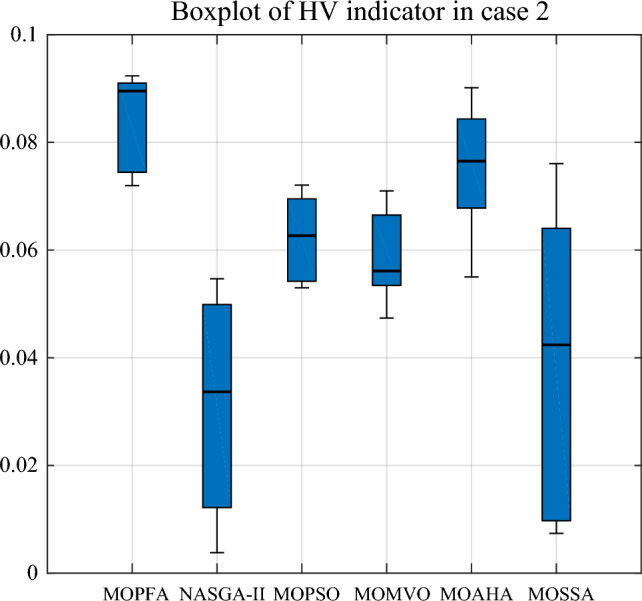
Boxplot of HV indicator in Case 2.

**Figure 9:**
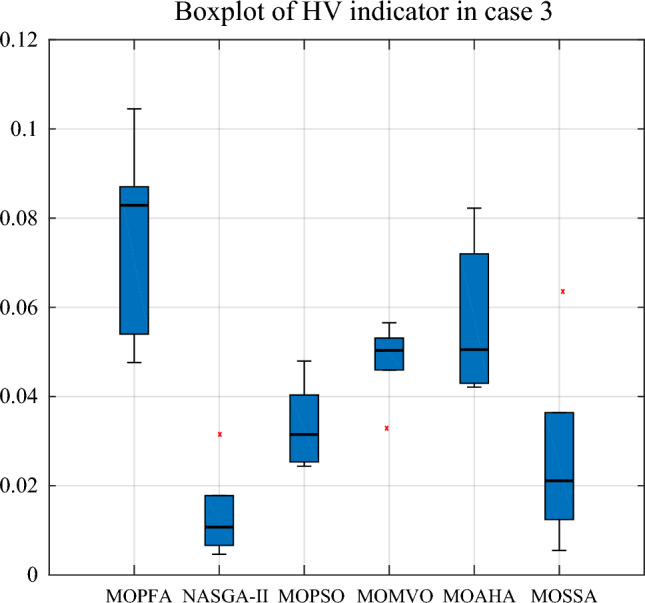
Pareto front in Case 3.

**Figure 10:**
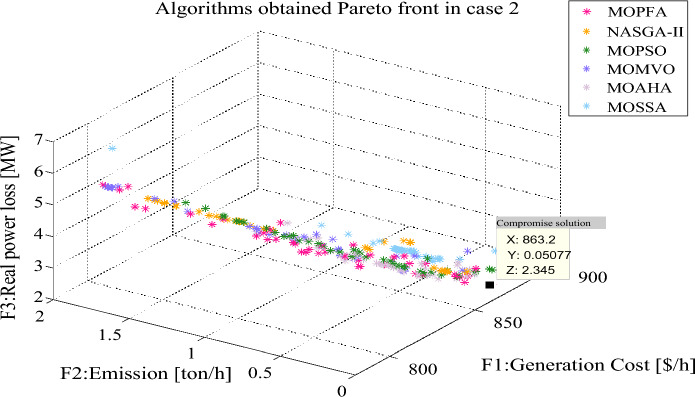
Pareto front in Case 2.

**Figure 11:**
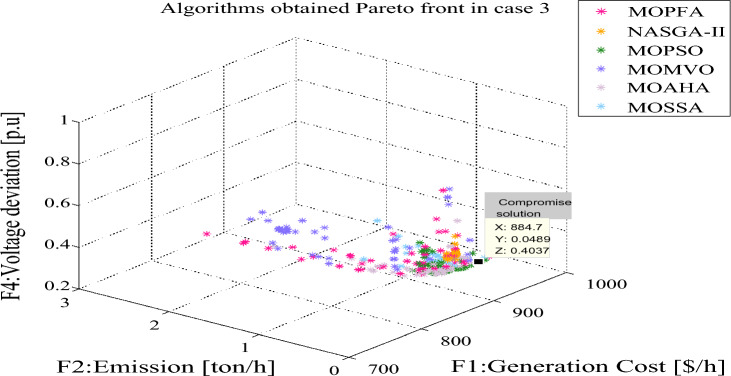
Pareto front in Case 3.

In Table 9, for Cases 2 and 3, the number of optimization objectives is three. Case 2's goal is to reduce generation costs, emissions, and real power loss. Case 3 aims to minimize generation cost, emission and voltage deviation. In Case 2 and Case 3, MOPFA obtained the maximum values of HV index 0.108043 and 0.133712 in the six algorithms and also achieved the best results on the minimum and average values of the HV indicators. The Wilkerson rank sum test scores of MOPFA in Cases 2 and 3 were 5.40 and 5.93, respectively, MOPFA ranked first among six algorithms in the two cases. The boxplot figure of the HV indicator obtained by each algorithm in cases 2 and 3 is given in Figs. 8 and 9, the variance of MOPFA is very small, indicating that the solution obtained by MOPFA is very stable. The Pareto front, resulting from the maximum run of the HV indicator is plotted in Figs. 10 and 11, it is obvious that the Pareto front obtained by MOPFA in Cases 2 and 3 is more evenly distributed than that obtained by other algorithms.

**Table 9:** HV-indicator in Case 2 and Case 3. Significant values are in bold.

Case	Algorithm	HV					
		Max	Min	Mean	Std	Score	Rank
Case 2	MOPFA	**0.108043**	**0.064170**	**0.084408**	0.010844	**5.40**	**1**
	NASGA	0.054673	0.000922	0.015265	0.015585	1.10	6
	MOPSO	0.088829	0.048563	0.062856	0.009252	3.53	4
	MOMVO	0.084535	0.042549	0.061788	0.011016	3.70	3
	MOAHA	0.095046	0.054996	0.077686	**0.011747**	5.00	2
	MOSSA	0.076067	0.007374	0.038917	0.018555	2.27	5
Case 3	MOPFA	**0.133712**	**0.047613**	**0.083691**	0.019008	**5.93**	1
	NASGA	0.031523	0.001726	0.010767	0.007625	1.76	6
	MOPSO	0.049571	0.020964	0.031593	0.007267	2.60	4
	MOMVO	0.085249	0.031574	0.048198	0.012535	3.93	3
	MOAHA	0.105672	0.024056	0.054976	**0.017598**	5.00	2
	MOSSA	0.063557	0.004595	0.022643	0.016286	1.77	5

**Figure 12:**
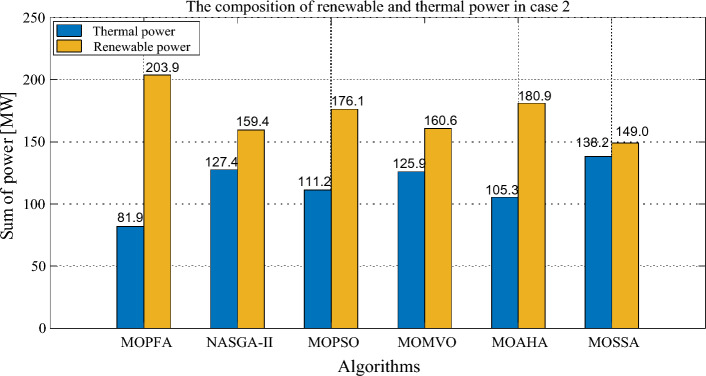
The composition of renewable and thermal power in Case 2.

**Figure 13:**
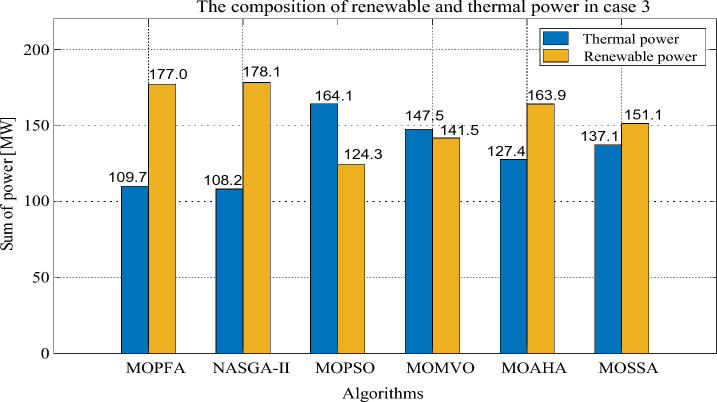
The composition of renewable and thermal power in Case 3.

The compromise solutions obtained by each algorithm in the two cases are given in Tables 10 and 12, respectively. In Case 2, the compromise solution obtained by MOPFA is marked in Fig. 10, the value of generation cost is 863.2328 ($/h), emission is 0.0508 (t/h) and real power loss is 2.3446. In Case 3, the compromise solution obtained by MOPFA is marked in Fig. 11, the value of the generation cost is 884.6613 ($/h), emission is 0.0489 (t/h) and the voltage deviation is 0.4037. Figures 12 and 13 provided the composition of renewable generation energy and thermal generation energy in the solutions obtained by each algorithm.

**Table 10:** Compromise solution values obtained by each algorithm. Significant values are in bold.

	LB	UB	Algorithm					
			MOPFA	NASGA-II	MOPSO	MOMVO	MOAHA	MOSSA
Control variables								
$$P_{{G_{2} }} \;({\text{MW}})$$	20	80	28.6566	54.4651	44.2135	61.6351	53.2922	55.3038
$$P_{{G_{5} }} \;({\text{MW}})$$	0	75	74.5949	60.5986	63.4783	60.3973	59.9942	59.9810
$$P_{{G_{8} }} \;({\text{MW}})$$	0	40	24.3144	22.9219	23.6391	21.9237	24.4547	21.6535
$$P_{{G_{11} }} \;({\text{MW}})$$	0	60	57.8024	41.9632	48.1911	48.0717	57.8974	34.9888
$$P_{{G_{13} }} \;({\text{MW}})$$	0	50	47.2424	33.9319	40.8802	30.2714	38.6188	32.3837
$$V_{1} \;({\text{P.U.}})$$	0.95	1.10	1.0428	1.0653	1.0073	1.0551	1.0624	1.0464
$$V_{2} \;({\text{P.U.}})$$	0.95	1.10	1.0514	1.0575	1.0296	0.9500	1.0297	1.0368
$$V_{5} \;({\text{P.U.}})$$	0.95	1.10	1.0779	1.0676	1.0897	1.0336	1.0309	1.0572
$$V_{8} \;({\text{P.U.}})$$	0.95	1.10	1.0478	1.0399	1.0488	1.1000	1.0453	1.0390
$$V_{11} \;({\text{P.U.}})$$	0.95	1.10	1.0828	1.0162	1.0898	1.1000	1.0527	1.0718
$$V_{13} \;({\text{P.U.}})$$	0.95	1.10	1.0997	1.0651	1.0779	1.1000	1.0223	1.0655
State variables								
$$P_{{G_{1} }} \;({\text{MW}})$$			53.3042	72.9842	67.0141	64.3562	52.0941	82.9726
$$Q_{{G_{1} }} \;({\text{MVAr}})$$			− 20	0.2124	− 20	9.8130	28.9735	− 3.6311
$$Q_{{G_{2} }} \;({\text{MVAr}})$$			13.5855	15.9054	2.0945	− 20	− 20	− 8.8179
$$Q_{{G_{5} }} \;({\text{MVAr}})$$			26.6269	24.3422	35	35	35	35
$$Q_{{G_{8} }} \;({\text{MVAr}})$$			40	40	40	31.4350	34.9244	40
$$Q_{{G_{11} }} \;({\text{MVAr}})$$			24.8168	4.2196	30	30	21.5755	23.7276
$$Q_{{G_{13} }} \;({\text{MVAr}})$$			25	25	25	25	12.1666	25
Objectives								
F1: Generation cost [$/h]			863.2328	838.6775	845.0190	847.8680	857.7920	**834.2038**
F2: Emission [ton/h]			0.0508	0.0732	0.0622	0.0601	**0.0482**	0.1024
F3: Real power loss [MW]			**2.3446**	3.4651	3.1340	3.2564	2.9515	3.8834

From Table 10, it can be seen that MOPFA achieved the best real power loss of 2.3446 MW, while the second-best score of 2.9515 MW was obtained by MOAHA, MOPFA's solution results in a 30% reduction in power loss compared to MOAHA's solution. MOSSA obtained the best generation cost value 834.2038 ($/h) in Case 2. MOAHA obtained the best emission value 0.0482 (ton/h) in Case 2. It can be seen from Fig. 11, MOPFA uses 81.9 MW thermal power generation energy, which is much lower than the 127.4 MW, 111.2 MW, 125.9 MW, 105.3 MW and 138.2 MW of other algorithms. In particular, wind farms, photovoltaic plants and tidal power plants connected to buses 5, 8, 11 and 13 are scheduled to be assigned 74.5949 MW, 57.8024 MW and 47.2424 MW respectively. This is more than 90% of the intended capacity of these renewable power plants, which will undoubtedly increase the default and reserve costs, but as the experimental data shows, the renewable energy generation process does not require fuel costs, so the cost increase is acceptable. The Case 2 results obtained by MOPFA were compared with the recently published literature in Table 11. The comparison results show that MOPFA obtained the minimum emission value 0.0508 (ton/h) than other algorithms. Although MOPFA’s power loss value of 2.1891 (ton/h) is inferior to TLBO’s^38^, the cost and emission of MOPFA are significantly lower than TLBO’s^38^ among the three targets. MOPFA’s emission and power loss is lower than NASGA-II-SF^30^, MOEA/D-SF^33^, PSO-SSO^34^, MOAGDE^31^ and ACNSDE-SF^30^. Different from Cases 2 and 3 adopts voltage deviation instead of power loss optimization target. From Table 12, it can be seen that MOPFA provides the best result on the emission (0.0489) and VD (0.4037), while MOMVO obtained the best cost value of 793.6699. Although MOPFA’s cost value is 884.6613 and it beyond other algorithms, carefully scrutinize Fig. 12, the reason is that MOPFA only uses 109.7 MW thermal power, while MOPSO uses 164.1 MW. In order to satisfy the power demand of the system, MOPFA dispatched more renewable energy supply, including 28.8977 MW of tidal energy and 74.3827 MW of wind energy, while MOPFA ensuring the minimization of VD and achieving the result of a minimum VD value. It is concluded that the proposed MOPFA can solve MOPF problems in a greener way and provide more diverse solutions for engineers to choose from.

**Table 11:** The compromise solution of Case 2 obtained by MOPFA was compared with the published literature.

Algorithm	Generation cost [$/h]	Emission [ton/h]	Real power loss [p.u]
MOPFA	863.2328	0.0508	2.3446
NASGA-II-SF^34^	853.54	0.11812	2.8492
TLBO^52^	882.2742	0.1004	2.1891
MOEA/D-SF^49^	881.012	0.2164	4.1441
PSO-SSO^50^	865.18	0.224	4.093
MOAGDE^4^	821.8398	0.2536	9.9646
ACNSDE-SF^34^	827.33	0.19659	4.1918

**Table 12:** Compromise solution values obtained by each algorithm. Significant values are in bold.

	LB	UB	Algorithm					
			MOPFA	NASGA-II	MOPSO	MOMVO	MOAHA	MOSSA
Control variables								
$$P_{{G_{2} }} \;({\text{MW}})$$	20	80	57.4243	51.9143	80	21.5395	61.4589	58.7567
$$P_{{G_{5} }} \;({\text{MW}})$$	0	75	74.3827	61.7280	63.3067	52.1651	53.7995	62.3518
$$P_{{G_{8} }} \;({\text{MW}})$$	0	40	34.1595	17.0056	35	20.8785	30.8963	23.5868
$$P_{{G_{11} }} \;({\text{MW}})$$	0	60	39.6052	49.5539	26.0829	33.9943	49.5359	46.6194
$$P_{{G_{13} }} \;({\text{MW}})$$	0	50	28.8977	49.8478	0	34.5303	29.6939	18.5482
$$V_{1} \;({\text{P.U.}})$$	0.95	1.10	1.0471	1.0557	1.0614	1.0258	0.9938	1.0119
$$V_{2} \;({\text{P.U.}})$$	0.95	1.10	1.0819	0.9676	1.0931	1.0323	1.0497	1.0421
$$V_{5} \;({\text{P.U.}})$$	0.95	1.10	1.0807	1.0481	1.0326	1.0771	1.0890	1.0405
$$V_{8} \;({\text{P.U.}})$$	0.95	1.10	0.9500	1.0873	0.9500	1.0998	0.9916	1.0081
$$V_{11} \;({\text{P.U.}})$$	0.95	1.10	1.0998	1.0840	1.0961	1.0999	1.0790	1.0771
$$V_{13} \;({\text{P.U.}})$$	0.95	1.10	1.0797	1.0787	1.1000	1.1000	1.0734	1.0859
State variables								
$$P_{{G_{1} }} \;({\text{MW}})$$			52.3740	56.3713	84.1607	125.9656	65.9542	78.3861
$$Q_{{G_{1} }} \;({\text{MVAr}})$$			− 13.5846	5.2632	− 6.9106	− 20	− 20	− 20
$$Q_{{G_{2} }} \;({\text{MVAr}})$$			60	− 20	60	6.4275	54.3186	35.1202
$$Q_{{G_{5} }} \;({\text{MVAr}})$$			− 30	35	− 30	35	− 13.4506	5.4266
$$Q_{{G_{8} }} \;({\text{MVAr}})$$			40	40	40	40	40	40
$$Q_{{G_{11} }} \;({\text{MVAr}})$$			30	25.7018	30	30	27.5689	27.7867
$$Q_{{G_{13} }} \;({\text{MVAr}})$$			25	25	25	25	25	25
Objectives								
F1: Generation cost [$/h]			884.6613	854.9569	794.2250	**793.6699**	876.0810	860.6207
F2: Emission [ton/h]			**0.0489**	0.0509	1.9315	0.9777	0.0622	0.0874
F4: Voltage deviation [p.u]			**0.4037**	0.4836	0.4094	0.4159	0.4075	0.4058

#### Result on Case 4: minimize the four objective functions

**Figure 14:**
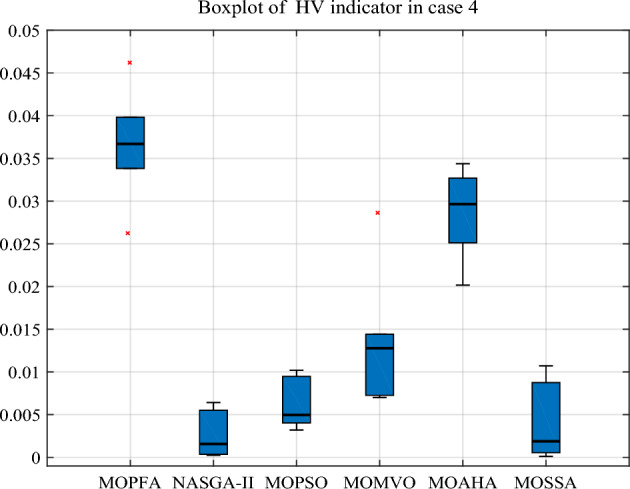
Boxplot of HV indicator in Case 4.

**Figure 15:**
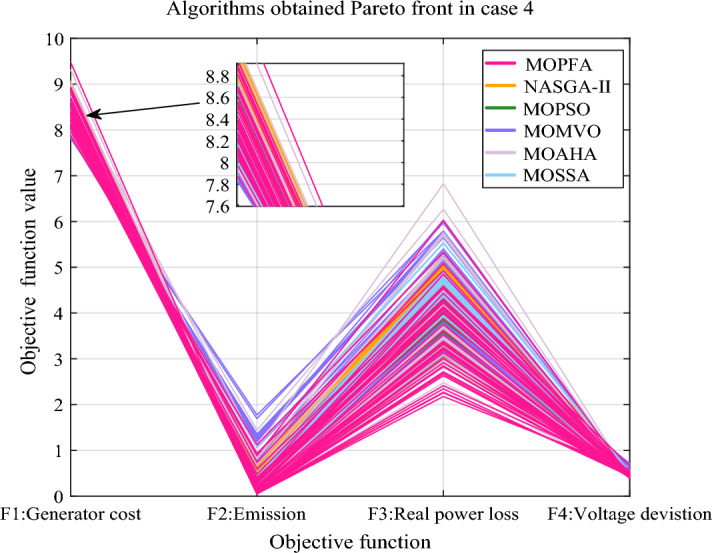
Pareto front in Case 4.

Earlier in the article, this research identifies four optimization objectives: generation cost, emission, real power loss and voltage deviation. In Case 4, four optimization objectives were selected together, this presents a big challenge to the optimization algorithm, but MOPFA still gives competitive results. The HV indicators obtained by MOPFA and other five algorithms after 30 runs are listed in Table 13. MOPFA achieves the best maximum, minimum and average values, the HV indicator variance plots of each algorithm are plotted in Fig. 14. MOPFA's results are remarkably stable and better than those of the other five algorithms, the results of the 30 runs were ranked by Wilkerson rank sum and MOPFA ranked first in six algorithms, it’s score is 5.88. Figure 15 shows the best Pareto front obtained by each algorithm. From Fig. 15, it can be seen that the distribution of Pareto optimal solutions of MOPFA on each objective is very uniform, especially on the third objective function F3: real power loss, which can be almost evenly distributed in the entire value range.

**Table 13:** HV-indicator in Case 4. Significant values are in bold.

Case	Algorithm	HV					
		Max	Min	Mean	Std	Score	Rank
Case 4	MOPFA	**0.058931**	**0.024535**	**0.039264**	0.0081685	**5.88**	**1**
	NASGA-II	0.014293	2.5373E−04	0.002985	0.0031157	1.77	5
	MOPSO	0.010190	0.001639	0.005150	**0.0023695**	2.58	4
	MOMVO	0.029612	0.004936	0.016069	0.0071498	3.75	3
	MOAHA	0.045301	0.013464	0.027674	0.0751510	5.10	2
	MOSSA	0.010712	1.2139E−04	0.003324	0.0027382	1.76	6

**Figure 16:**
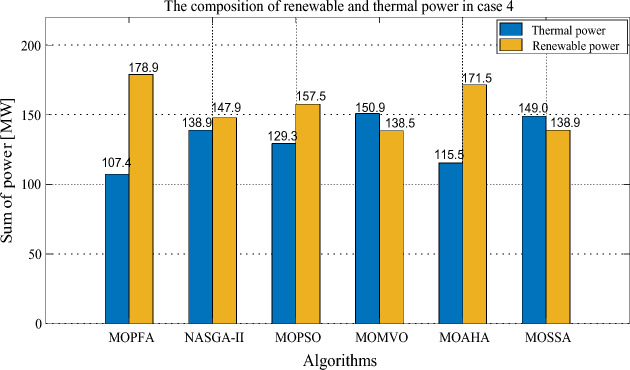
The composition of renewable and thermal power in Case 4.

The selected compromise solutions of six algorithms in this Case are reported in Table 14. According to this table, MOPFA's solution has the lowest emission: 0.0486 (ton/h) and the highest real power loss (3.0052). MOMVO obtained the minimum value for generation cost, but the value of emission and real power loss are more than twice as high as MOPFA’s. Through the energy composition histogram in Fig. 16, the reason for the compromise solution is analyzed. Because MOPFA's solution dispatches the most tidal energy, this may be because tidal power is more stable than wind power, reducing the extra cost of renewables to some extent. The total renewable power of MOPFA’s solution is 178.9 MW, it is bigger than NASGA-II: 147.9 MW. But dispatching through MOPFA increases the use of more stable tidal energy in solutions, which reduces the uncertainty of wind and photovoltaic energy. As a result, MOPFA’s generation costs 5.6% less than NASGA-II. Table 15 reports a comparison of the solutions obtained by MOPFA and the recently published literature. From Table 15, it is obvious that MOPFA gets the smallest emission (0.0486), PSO-SSO^50^ gets the smallest cost, NASGA-II-SF^34^ gets the best voltage deviation. The solutions provided by these algorithms do not dominate each other, but the MOPFA solution should be considered when considering emissions taxation. In summary, MOPFA offers a more diverse set of solutions, and MOFPA offers solutions to increase the use of more stable tidal energy when renewable energy uncertainty increases the cost of the solution. This will help to resolve the contradiction between costs and emissions, and further demonstrates that the use of three renewable energy sources connected to the grid can greatly reduce emissions, and this energy composition will be very promising in the future.

**Table 14:** Compromise solution values obtained by each algorithm. Significant values are in bold.

	LB	UB	Algorithm					
			MOPFA	NASGA-II	MOPSO	MOMVO	MOAHA	MOSSA
Control variables								
$$P_{{G_{2} }} \;({\text{MW}})$$	20	80	55.0827	67.7103	57.3602	21.8571	22.5218	47.7580
$$P_{{G_{5} }} \;({\text{MW}})$$	0	75	51.5612	71.7705	61.5017	38.9172	66.2482	46.7818
$$P_{{G_{8} }} \;({\text{MW}})$$	0	40	22.5658	28.8133	25.0488	22.7267	21.2179	28.1285
$$P_{{G_{11} }} \;({\text{MW}})$$	0	60	60	38.0716	38.3362	42.6629	41.4882	32.9828
$$P_{{G_{13} }} \;({\text{MW}})$$	0	50	44.8031	9.2449	32.6630	34.2024	42.5803	31.0533
$$V_{1} \;({\text{P.U.}})$$	0.95	1.10	1.0593	1.0591	1.0400	1.0797	1.0456	1.0547
$$V_{2} \;({\text{P.U.}})$$	0.95	1.10	1.0398	1.0595	1.0353	0.9658	1.0187	1.0456
$$V_{5} \;({\text{P.U.}})$$	0.95	1.10	1.0557	1.0560	1.0794	1.0998	1.0979	1.0926
$$V_{8} \;({\text{P.U.}})$$	0.95	1.10	1.0999	1.0704	1.0356	0.9907	1.0303	1.0162
$$V_{11} \;({\text{P.U.}})$$	0.95	1.10	1.0891	1.0813	1.0923	1.0999	1.0780	1.0786
$$V_{13} \;({\text{P.U.}})$$	0.95	1.10	1.0684	1.0217	1.0705	1.0707	1.0959	1.0727
State variables								
$$P_{{G_{1} }} \;({\text{MW}})$$			52.3824	71.2731	71.9532	129.0667	93.0083	101.2521
$$Q_{{G_{1} }} \;({\text{MVAr}})$$			7.1748	− 16.8915	− 13.1505	50.4159	6.7013	− 7.6273
$$Q_{{G_{2} }} \;({\text{MVAr}})$$			− 20	18.8164	− 5.2117	− 20	− 20	17.2120
$$Q_{{G_{5} }} \;({\text{MVAr}})$$			35	35	34.2552	− 5.6715	33.0471	13.7113
$$Q_{{G_{8} }} \;({\text{MVAr}})$$			40	40	40	40	40	40
$$Q_{{G_{11} }} \;({\text{MVAr}})$$			27.7525	25.3068	30	30	27.4323	24.9460
$$Q_{{G_{13} }} \;({\text{MVAr}})$$			23.0593	7.9208	25	25	25	25
Objectives								
F1: Generation Cost [$/h)			852.2493	903.5947	841.0084	**790.0268**	828.3344	823.4770
F2: Emission [ton/h]			**0.0486**	0.0726	0.0715	1.1849	0.1598	0.2340
F3: Real power loss [MW]			**3.0052**	3.4297	3.4722	6.0329	3.6648	4.5565
F4: Voltage deviation [p.u]			0.5193	0.4388	**0.4111**	0.4158	0.4310	0.4147

**Table 15:** The compromise solution of Case 4 obtained by MOPFA was compared with the published literature. Significant values are in bold.

Algorithm	Generation cost [$/h]	Emission [ton/h]	Real power loss [MW]	Voltage deviation [p.u]
MOPFA	852.2493	**0.0486**	3.0052	0.5193
NASGA-II-SF^34^	845.32	0.42792	4.2069	**0.39792**
TLBO^52^	878.3400	0.0958	**2.6208**	0.4528
MOEA/D-SF^49^	919.04	0.6221	5.5429	0.4530
PSO-SSO^50^	**826.94**	0.258	5.5150	0.466
MOAGDE^4^	826.5070	0.2227	9.4052	0.8141
ACNSDE-SF^34^	837.46	0.18045	3.6984	0.4179

#### System constraints of the best compromise solution

**Figure 17:**
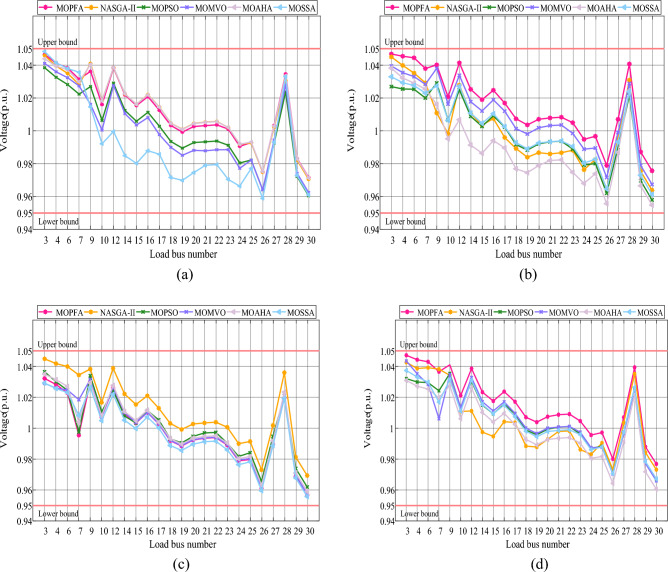
Different load bus voltage of the best compromise solutions obtained by six algorithms on IEEE 30-bus system: (**a**) Case 1, (**b**) Case 2, (**c**) Case 3, (**d**) Case 4.

In the MOOPF problem, the voltage on each branch must be within the secure range to guarantee the power system's regular functioning. Because the bus containing the green energy units and thermal power units is already in the range, the voltage on the remaining 24 buses must be in the range of [0.95 p.u., 1.05 p.u.]. As a result, in order to validate the effectiveness of the answer achieved by each algorithm in Cases 1–4, Fig. 17 depicts the voltage of each branch of each algorithm on the IEEE 30-bus in the four study cases, clearly showing:

In Cases 1–4, the intermediate solution produced by six algorithms meets the branch voltage restriction, demonstrating the possibility of using a meta-heuristic algorithm to solve the MOOPF issue. When we concentrate on Case 1, each algorithm's obtained voltage in bus 3 is near to, but not exceeding, the upper bound. The voltage intervals of each algorithm are not noticeably different in the other three testing instances. In summary, each calculation's compromise answer meets the branch voltage limit.

## Conclusion and future work

In this work, a multi-objective optimization power flow (MOOPF) problem with stochastic wind, solar power and tidal power models is introduced. Different scenarios of renewable energy supply are simulated by considering different probability distribution functions. To solve this complex multi-objective optimization problem, a novel multi-objective optimization algorithm MOPFA was proposed. The thermal power generators in IEEE 30-bus were replaced by wind turbines, photovoltaic power plants, and tidal power generation equipment. The simulation is carried out on the modified IEEE 30-bus system. The experiment was conducted in four different cases and the experimental results are compared with those of other well-known multi-objective optimization algorithms. Statistical results showed that MOPFA achieved the best HV indicator in all four cases. In addition, MOPFA's Wilkerson rank sum test was also ranked first, while MOPFA is slightly better than other algorithms. In solve the multi-objective optimal power flow problem, MOPFA can obtain a more widely distributed solution set, and the solution that satisfies the constraints, so it can be used as the preferred algorithm to solve this problem. The compromise solution is calculated from the solution set obtained by MOPFA by fuzzy logic, and the compromise solution of MOPFA uses more renewable energy supply and effectively reduces emissions. Incorporating renewable energy into the power system can reduce emissions while maintaining system stability, which will be an advantage in the future, so the compromise solution obtained by MOPFA is more in line with the development trend of future power dispatch. In the future, this research will focus on the direction of solving the MOOPF problem on larger IEEE test systems with real wind energy datasets, and search for real power price datasets from the government to build more accurate models.

## Data Availability

The datasets used and analysed during the current study available from the corresponding author on reasonable request.
